# Society Meeting

**Published:** 1896-03

**Authors:** 


### Society Meeting.

The Buffalo Ophthalmological Society was organised last month for the purpose of studying and discussing ophthalmological subjects and cases.

Its constitution provides for regular and associate members. Regular members must have had at least five years of ophthalmic practice and shall consist of the original members and such as are elected from the associate membership. The associate members must have had at least three years of ophthalmic practice, and are entitled to all the privileges of the society, except to vote and hold office.

Meetings will be held on the third Monday evening of each month for eight months of each year, beginning October.

The first regular meeting was held at the office of Dr. Abbott, the senior ophthalmologist of Buffalo, February 17, 1896. Dr. Howe described a self-retaining fixation forceps; Dr. Hubbell reported a case of congenital growth from the right inner canthus, and Dr. Starr related some of his experiences in "cathode" photography.

The next meeting will be held Monday evening, March 18, 1896.

